# Evaluation of the effect of intensity‐modulated radiotherapy (IMRT) and volumetric‐modulated arc radiotherapy (VMAT) techniques on survival response in cell lines with a new radiobiological modeling

**DOI:** 10.1002/cam4.6593

**Published:** 2023-09-27

**Authors:** Serra Kamer, Sunde Yilmaz Susluer, Tugce Balci Okcanoglu, Cagla Kayabasi, Besra Ozmen Yelken, Sinan Hoca, Emin Tavlayan, Nezahat Olacak, Yavuz Anacak, Murat Olukman, Cumhur Gunduz

**Affiliations:** ^1^ Department of Radiation Oncology Ege University Medical Faculty Izmir Turkey; ^2^ Department of Medical Biology Ege University Medical Faculty Izmir Turkey; ^3^ Department of Pharmacology Ege University Medical Faculty Izmir Turkey

**Keywords:** cell survival, IMRT, radiotherapy techniques, VMAT

## Abstract

**Background:**

The optimal radiobiological model, which assesses the biological effects of novel radiotherapy techniques that concurrently modify multiple physical factors, has not yet been defined. This study aimed to investigate the impact of intensity‐modulated radiotherapy (IMRT) and volumetric‐modulated arc therapy (VMAT) on cellular response in head and neck cancer and melanoma models.

**Methods:**

Clonogenic analysis, DNA double‐strand break analysis, apoptosis, and cell cycle analysis were performed on cancer stem cell models, cancer models, and normal tissue cell models to assess radiation sensitivity.

**Results:**

The segmented radiation approach used in IMRT applications enhanced radiosensitivity and cytotoxicity in the cancer models, while changes in dose rate had varying effects on cytotoxicity depending on the tumor cell type. VMAT increased cellular resistance, favoring treatment outcomes.

**Conclusions:**

The biological processes were influenced differently by dose rate, IMRT, and VMAT depending on the tumor cell type. The selection of the most appropriate technique is crucial in representing new radiotherapy approaches. The obtained data can serve as a model to address clinical questions in daily practice. The integration of non‐standard outcomes with standard applications should be considered in clinical settings.

## 1 INTRODUCTION

Cancer is a significant health concern for adults and is second only to cardiovascular diseases. Among the primary treatment modalities for cancer patients, radiotherapy plays a vital role.^1^ Radiotherapy is a technique employed in the management of cancer. Its primary objective is to administer the highest feasible dose to a detected tumor mass, while minimizing harm to the surrounding healthy tissue. This approach aims to preserve the patient's quality of life while maximizing the likelihood of controlling and curing the cancer.^2^ Advancements in computer technology have also resulted in enhancements in treatment planning systems. In the past, dose distributions could only be computed in the transverse section. However, with three‐dimensional reconstructions from these sections, it is now feasible to calculate dose distributions in the sagittal and coronal sections as well. While the intensity‐modulated radiotherapy (IMRT) technique enhances the tumor's dose delivery, it simultaneously reduces the dose received by the surrounding tissues. Conversely, IGRT technology offers valuable insights into the actual state of the tumor and organs by capturing three‐dimensional images prior to treatment.^3^ The biological effects of ionizing radiation have been established through radiobiological research, which forms the basis for clinical calculations of radiotherapy doses.^4^ However, while there have been technological advancements in radiation therapy, basic research in radiobiology has not progressed at the same pace, highlighting the need for a new radiobiological system that can accurately simulate changes in various physical dose parameters.^5^ Blockhuys et al. summarized the radiobiological questions associated with intensity‐modulated radiotherapy techniques. The review emphasized the presence of unanswered questions in clinical applications and provided suggestions regarding radiobiological modeling.^6^ It suggests the creation of new modeling approaches for in vitro studies of intensity‐modulated radiotherapy and identifies differences in cellular survival between different cell types. While the impact of treatment duration on cellular survival has been demonstrated, the description of techniques that shorten the duration by modifying dose rate within the treatment has been insufficient.^7^ The step‐and‐shot technique, which represents the initial clinical implementation of intensity‐modulated radiotherapy, was investigated by Butterworth et al. in three different cell types. The modeling was applied as segmented dose distribution in a monolayer flask. A simplified model of daily practice was created, and it was revealed that an extended duration had an impact on cellular survival.^8^ Furthermore, cancer stem cells (CSCs) have a significant involvement in the failure of radiotherapy.^9^

The main objective of this study was to develop a radiobiology model that accurately reproduces the biological effects of advanced radiation procedures. Previous literature has identified the lack of a suitable model for these state‐of‐the‐art techniques. Therefore, our investigation focused on assessing the cellular response to complex dose distributions generated using the IMRT approach, considering various physical factors. Our ultimate goal was to establish a model that closely reflects the daily applications of these techniques.

In this study, we conducted a comprehensive examination of the impact of parameters commonly used in everyday clinical practice, utilizing novel technological devices, on in vitro cell survival. To simulate real‐life scenarios, we employed three distinct cell models: two tumor cell lines known for their robust radioresistance properties, tumor stem cell lines recognized as the primary contributors to radiotherapy resistance, and normal cell lines susceptible to the effects of radiotherapy. After establishing appropriate dose ranges, we meticulously investigated the effects of dose, dose rate, and non‐uniform dose distribution on cellular survival using various assays, including clonogenic assay, DNA double‐strand break assay, apoptosis assay, cell cycle assay, and autophagy assay. Additionally, we explored the in vitro effects of volumetric‐modulated arc therapy (VMAT) application using a phantom that closely mimics daily clinical applications. Through this comprehensive study, our aim was to address the existing gap in the literature by developing an appropriate radiobiology model for advanced radiation techniques. The findings from our research contribute to a better understanding of the cellular response to complex dose distributions and have significant implications for improving treatment planning and enhancing patient outcomes in clinical practice.

## 2 MATERIALS AND METHODS

### 2.1 VMAT irradiation model

**FIGURE 1:**
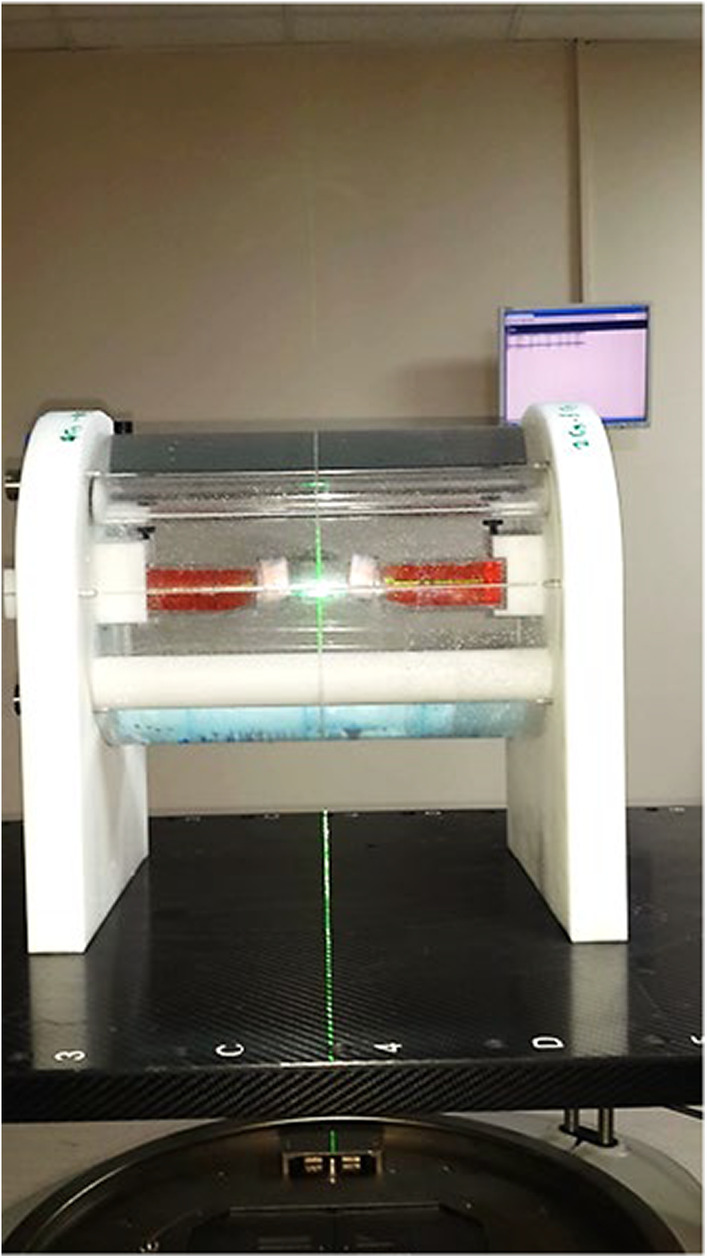
The image illustrates a water phantom that is employed for simulating VMAT (volumetric‐modulated arc therapy) irradiation. It provides a physical environment that allows for the examination and assessment of dose distributions and their impact on cellular response. The water phantom, with its cylindrical shape, serves as a suitable medium for mimicking the irradiation conditions encountered during VMAT treatment. This setup enables researchers to study the effects of complex dose distributions on tumor tissue and normal tissues, thereby contributing to a better understanding of the radiobiological aspects associated with VMAT irradiation.

A cylindrical water phantom was specifically designed for the VMAT irradiation technique to accommodate T25 cell culture flasks, allowing for the simultaneous irradiation of two flasks. The phantom was designed to maintain physical conditions similar to the VMAT technique, ensuring reproducible irradiation setups. To mimic different density levels of the human body and create a scenario similar to daily applications, materials with four different densities were placed inside the phantom. When the phantom was filled with water (density of 1 g/cm^3^), it provided a complete scattering medium with varying density levels for irradiating the flasks (Figure 1). Cross‐sectional images required for calculating the three‐dimensional radiation dose distributions (2, 4, 6, and 8 Gy) delivered by the VMAT technique to the cell flasks inside the water phantom were obtained through computerized tomography. These images were then transferred to the planning system (Monaco 3.2). The planning target volume (PTV) was defined around the flask with a 5 mm margin, ensuring that the PTV received at least 95% of the targeted dose values (in Gy). Before irradiating the cells, an ion chamber (0.125 cm^3^, PTW SemiFlex) was used to verify the calculated monitor unit (MU) values, ensuring that the dose values fell within ±3% of the planned values. Additionally, Gafchromic EBT3 films were placed at the bottom of each cell flask during irradiation to confirm the targeted doses using a secondary dosimetry method. The dose values obtained from the Gafchromic films also confirmed that the doses delivered to the flasks were within ±5%. All analyses were performed in triplicate.

### 2.2 Irradiation technique studies representing standard irradiation

#### 2.2.1 Cell culture

Human melanoma CSC line (36118‐45P, Celprogen) and human head and neck CSC line (36125‐52P, Celprogen) were utilized as CSC models. Additionally, a melanoma cell line (NM2C5/RRID:CVCL_B064) and head and neck cancer cell lines (A253−/RRID:CVCL_1060 and FADU/RRID:CVCL_1218) were established as cancer cell models. Fibroblast cell line (WI‐38/RRID:CVCL_0579) and epithelial cell line (WSS‐1/RRID:CVCL_2767) were employed as control models representing normal tissue cells. All cell lines were cultured in a standard cell culture incubator at 37°C, under a 5% CO_2_ atmosphere, and with 95% relative humidity. Routine testing for mycoplasma contamination was conducted using DAPI staining. The experiments were performed using cells between passages four to six.

#### 2.2.2 Clonogenic analysis

All cancer cell models and control cells were exposed to irradiation at doses of 2, 4, 6, 8, and 10 Gy for clonogenic assays. The untreated group consisted of 100 cells, while the 2 Gy treatment group had 200 cells, the 4 Gy treatment group had 600 cells, the 6 Gy treatment group had 1000 cells, the 8 Gy treatment group had 1200 cells, and the 10 Gy treatment group had 1400 cells. After 3 weeks of irradiation, colonies containing at least 50 cells (>1 mm) were evaluated in the clonogenic assay. All analyses were conducted in triplicate. Statistical analyses were performed using the SPSS program with the following procedures. For the quadratic term, “D2” was defined as the product of the dose and dose (D2 = dose × dose). For survival analysis, the variable “S” was calculated as the natural logarithm of the ratio of colonies to cells (ln(colonies/cells)) minus the natural logarithm of the plating efficiency (ln(PE)). The weight of observed colonies was calculated using the formula “W” = colony × cells/(cells−colony). Linear regression analysis was conducted using the SPSS software (Analyze ‐> Regression ‐> Linear). The dependent variable “S” was used, while the independent variables “dose,” “D2,” and “W” (under WLS in SPSS) were included. The analysis included confidence intervals and R‐squared values (Statistics in SPSS). Since the regression line passes through the origin, the option to include a constant in the equation (Options in SPSS) was not selected.^10^ Survival curves were generated by applying the linear‐quadratic regression model using GraphPad Prism software, with the fraction of surviving cells (Y) represented. The α/β ratio was calculated using the equation: α/β = exp[−(αD + βD2)], where D is the radiation dose (Gy). From the regression curves, D0 and D10 values were obtained using GraphPad Prism software. D10 represents the dose inducing 90% cell death.^11^

#### 2.2.3 Cell proliferation assay

Cells were seeded in 96‐well plates at an initial density of 1 × 10^4^ cells/100 μL. The cells were then exposed to various doses of ionizing radiation and incubated for 24, 48, and 72 h. Cell proliferation was assessed using the WST‐1 assay method, which involved adding the WST‐1 Cell Proliferation Reagent to each well, followed by measuring the absorbance at 450‐nm and 620‐nm wavelengths using a microplate reader. The effects of irradiation on cell proliferation were quantified by analyzing the dose–response curve using Sigmoidal dose–response curve analysis in the Calcusyn 2.0 software.

#### 2.2.4 Apoptosis analyzes

Cell lines were seeded in six microplates at a concentration of 5 × 10^5^ cells/mL and exposed to radiation doses ranging from 0 to 10 Gy. Annexin V (Annexin V‐EGFP Apoptosis Detection Kit‐Biovision) and Mitoprobe JC‐1 (MitoScreen JC‐1 kit‐BD Biosciences) methods were employed to study apoptosis in three replicates, following the manufacturer's instructions. The untreated group served as the control group. Flow cytometry analysis was conducted using the BD Acuri 6 system to evaluate the results.

#### 2.2.5 DNA double‐strand break analysis

The impact of varying radiation doses on DNA double‐strand breaks in cancer models was examined using γ‐H2AX analysis (Alexa Fluor 488 Mouse anti‐H2AX kit). Cell lines exposed to radiation doses ranging from 0 to 10 Gy were seeded at a concentration of 5 × 10^5^ cells/mL in six microplates. The analysis was performed at the 48th hour, a time point at which significant DNA double‐strand breaks could be detected. Flow cytometry was employed for evaluation.

#### 2.2.6 Cell cycle analysis

Cell lines were seeded at a concentration of 5 × 105 cells/mL in six microplates and exposed to radiation doses ranging from 0 to 10 Gy. The cell cycle distribution in cancer cells was investigated using cell cycle analysis (BD Cycletest Plus kit) at 24, 48, and 72 h after radiation exposure. Propidium iodide (PI) bound to DNA was quantified using flow cytometry with 488‐nm excitation and 586/42 bandpass emission filters. The FACSuite software was used to determine the number of cells in the sub G0/G1, G0/G1, S, G2/M, and >4n stages, and the percentages were calculated accordingly.

#### 2.2.7 Statistical analyses

Descriptive statistics were employed for data analysis. Nonlinear regression analysis based on the linear‐quadratic model was used to investigate the relationship between radiation doses and the survival fraction of cells. The same analysis was conducted to assess the impact on cell survival response using the phantom. A one‐way analysis of variance (ANOVA) was performed to compare the effects of different dose rates after 4 Gy irradiation. Apart from the aforementioned statistical analyses, all other statistical analyses were carried out using GraphPad Prism 9.5. The significance level was set at *p* < 0.05.

## 3 RESULTS

### 3.1 Results of irradiation technique representing standard irradiation

#### 3.1.1 Clonogenic analysis

##### Tumor cell models

**FIGURE 2:**
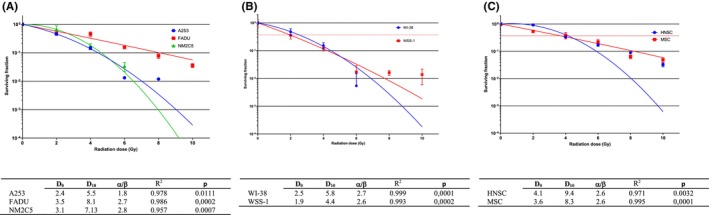
Presents the clonogenic analysis results for different cell models: (A) tumor cell model, (B) normal tissue model, and (C) stem cell model. Clonogenic analysis is a widely used technique to evaluate the survival and proliferation potential of cells after irradiation. In this study, the analysis was conducted to assess the response of each cell model to the experimental conditions. D_0_ and D_10_ are important parameters used to characterize the radiation response of cells. D_0_ represents the dose required to reduce the surviving fraction of cells to 37% (SF37), while D_10_ represents the dose required to reduce the surviving fraction to 10% (SF10). The α/β ratio is an important parameter used in radiobiology to characterize the response of cells to radiation. It represents the relative contributions of linear and quadratic components in the dose–response relationship. The α component reflects the linear component, which represents the cell killing effect proportional to the dose, while the β component represents the quadratic component, indicating the cell killing effect that increases with the square of the dose. Lower α/β ratio, indicating a greater dependence on the linear component, is more effectively treated with hypofractionation, which involves delivering higher doses per fraction. On the contrary, a higher α/β ratio, indicating a greater contribution from the quadratic component, is better managed with conventional fractionation, where lower doses per fraction are delivered over a longer treatment period. *R* ^2^, also known as the square of the correlation coefficient, is a statistical measure used to evaluate the goodness of fit of the dose–response curve. It provides information about the strength and direction of the relationship between the radiation dose and the survival fraction of cells.

In the head and neck cancer model, the coating efficiency was 17.20% in the A253 cell line and 40.33% in the FADU cell line. The melanoma cancer model (NM2C5 cell line) exhibited a coating efficiency of 36.33%. A significant correlation was observed between the dose and survival (*R* ^2^ = 0.978, *p* = 0.011, Figure 2A).

##### CSC models

Interestingly, the coating efficacy in the human (parental) head and neck CSC line was 99.67%. In contrast, the coating efficiency in the human (parental) melanoma CSC line was 66.33%. A significant correlation was observed between the dose and survival (*R* ^2^ = 0.914, *p* = 0.003, Figure 2C).

##### Normal tissue cell models

The WI‐38 fibroblast cell line exhibited a coating efficiency of 61.33%, while the WSS‐1 epithelial cell line had a coating efficiency of 29.33%. There was a significant correlation between the dose and survival (*R* ^2^ = 0.999, *p* < 0.001). The head and neck cancer model demonstrated higher radiosensitivity compared to the melanoma model. The WSS‐1 epithelial cell line and WI‐38 fibroblast cell line were chosen to represent early and late responding normal tissues in radiobiological modeling due to their distinct behavioral traits, accurately reflecting the characteristics of normal tissues. Notably, CSC models displayed different radiosensitivity properties in comparison with tumor cell models (Figure 2B).

#### 3.1.2 Cell proliferation assay

**FIGURE 3:**
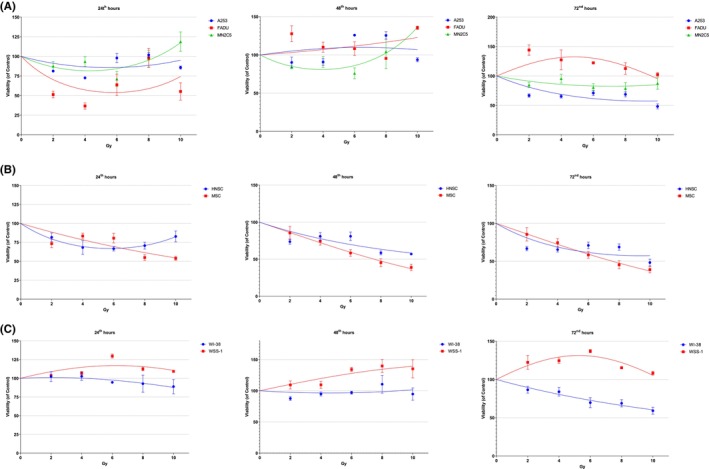
The radio‐cytotoxicity assay results for different cell models are presented: (A) tumor cell model, (B) stem cell model, and (C) normal tissue model. The radio‐cytotoxicity assay is a valuable experimental technique used to evaluate the cytotoxic effects of radiation on cells. It provides insights into the response of different cell types to radiation treatment.

In the A253 cell line, approximately 50% radio‐cytotoxicity was observed at 10 Gy after 72nd hours. Significant radio‐cytotoxicity was observed in the FADU cell line at 24th hours, while no radio‐cytotoxicity was observed at 48th and 72th hours. The NM2C5 cell line did not exhibit radio‐cytotoxicity at doses ranging from 2 to 10 Gy at 24th and 72nd hours (Figure 3A). In the human (parental) head and neck CSC line, radio‐cytotoxicity was observed at doses ranging from 2 to 10 Gy at 24th and 72nd hours, with approximately 50% radio‐cytotoxicity observed at 10 Gy after 72nd hours. The human (parental) melanoma CSC line displayed around 50% radio‐cytotoxicity at 8 and 10 Gy after 24th and 72nd hours (Figure 3B). In the WI‐38 cell line, approximately 50% radio‐cytotoxicity was observed at 10 Gy after 72nd hours. No radio‐cytotoxicity was observed in the WSS‐1 cell line at doses ranging from 2 to 10 Gy at 24th and 72nd hours (Figure 3C).

#### 3.1.3 Apoptosis analysis

##### Annexin V method

Apoptosis was observed in the A253 cell line after exposure to radiation doses of 2–10 Gy at 48th and 72nd hours. Similarly, in the FADU cell line, apoptosis was induced at 4–10 Gy after 72nd hours compared to the control group (0 Gy). Interestingly, apoptosis was not observed in the NM2C5 cell line when exposed to radiation doses of 2–10 Gy from 24th to 72nd hours. In the human (parent) head and neck CSC line, apoptosis was triggered at 2–10 Gy after 48th hours, and in the human (parental) melanoma CSC line, apoptosis occurred at 6–10 Gy after 72nd hours, both compared to the control group. Additionally, in the WI‐38 cell line, apoptosis was induced at 2–10 Gy after 24th hours, while in the WSS‐1 cell line, apoptosis was induced at 8 and 10 Gy from 24th to 72nd hours compared to the control group (Table 1).

**TABLE 1:** Fold changes in apoptosis detected with annexin V in cell lines compared to the control group.

		A253		FADU		MN2C5		HNSC		MSC		WI‐38		WSS‐1	
Hours	Gy	FC	*p*	FC	*p*	FC	*p*	FC	*p*	FC	*p*	FC	*p*	FC	*p*
24	2	1.61	<0.0001	0.49	<0.0001	0.75	0.0177	0.41	<0.0001	1.27	0.0094	1.82	<0.0001	1.44	<0.0001
	4	0.83	0.1611	0.90	0.6198	0.79	0.0573	0.44	<0.0001	1.49	<0.0001	2.41	<0.0001	1.64	<0.0001
	6	0.71	0.0049	0.76	0.024	1.39	0.0001	0.86	0.3116	1.53	<0.0001	2.57	<0.0001	5.28	<0.0001
	8	0.98	0.9989	0.61	0.0001	1.18	0.1263	0.65	0.0006	2.81	<0.0001	2.38	<0.0001	2.27	<0.0001
	10	0.14	<0.0001	0.61	0.0001	0.65	0.0006	0.82	0.1263	1.45	<0.0001	—	—	1.61	<0.0001
48	2	2.25	<0.0001	0.53	<0.0001	1.05	0.9589	3.10	<0.0001	0.84	0.2031	0.71	0.0047	0.60	0.0001
	4	2.97	<0.0001	2.09	<0.0001	0.91	0.7056	1.87	<0.0001	1.13	0.3784	0.90	0.5592	1.68	<0.0001
	6	1.41	<0.0001	0.89	0.5345	1.83	<0.0001	2.42	<0.0001	1.15	0.2531	1.54	<0.0001	1.27	0.0094
	8	1.45	<0.0001	1.75	<0.0001	1.07	0.8594	3.42	<0.0001	0.96	0.9838	1.43	<0.0001	2.04	<0.0001
	10	2.63	<0.0001	2.49	<0.0001	0.89	0.5345	3.65	<0.0001	1.04	0.9838	—	—	2.32	<0.0001
72	2	4.49	<0.0001	1.15	0.2531	1.05	0.9589	1.32	0.0018	1.68	<0.0001	0.26	<0.0001	0.65	0.0006
	4	3.80	<0.0001	2.37	<0.0001	1.30	0.0035	1.55	<0.0001	1.94	<0.0001	1.02	0.9973	2.26	<0.0001
	6	3.29	<0.0001	3.05	<0.0001	1.26	0.0129	1.68	<0.0001	2.39	<0.0001	1.46	<0.0001	2.35	<0.0001
	8	5.46	<0.0001	2.00	<0.0001	1.56	<0.0001	4.00	<0.0001	3.94	<0.0001	1.56	<0.0001	9.43	<0.0001
	10	3.97	<0.0001	3.78	<0.0001	1.34	0.0009	1.82	<0.0001	3.87	<0.0001	—	—	26.35	<0.0001

##### Mitoprobe JC‐1

Apoptosis was induced at 6–10 Gy at 48th and 72nd hours in the A253 cell line, and at 6–10 Gy at 72nd hours in the FADU cell line compared to the control group. In line with the annexin V results, apoptosis was not observed in the NM2C5 cell line at 2–10 Gy at 24th and 72nd hours compared to the control group. Apoptosis was induced at 2–10 Gy at 24th and 72nd hours in the human (parental) head and neck CSC line, and at 6–8 Gy at 72nd hours in the human (parental) melanoma CSC line compared to the control group. Consistent with the annexin V results, compared to the control group, apoptosis was induced at 2–10 Gy at 24th hours in the WI‐38 fibroblast cell line and induced at 6–10 Gy at 24th and 72nd hours in the WSS‐1 cell line (Table 2).

**TABLE 2:** Fold changes in apoptosis detected with JC‐1 in cell lines compared to the control group.

		A253		FADU		MN2C5		HNSC		MSC		WI‐38		WSS‐1	
Hours	Gy	FC	*p*	FC	*p*	FC	*p*	FC	*p*	FC	*p*	FC	*p*	FC	*p*
24	2	0.74	0.0129	0.95	0.9589	1.14	0.9589	2.47	<0.0001	0.19	<0.0001	1.89	<0.0001	1.36	0.0004
	4	0.78	0.0432	1.72	<0.0001	1.00	<0.0001	0.53	<0.0001	0.48	<0.0001	1.98	<0.0001	0.96	0.9838
	6	0.41	<0.0001	1.28	0.0068	2.00	0.0068	1.03	0.9955	0.43	<0.0001	3.81	<0.0001	2.12	<0.0001
	8	0.82	0.1263	1.35	0.0006	1.71	0.0006	5.53	<0.0001	1.14	0.3116	6.18	<0.0001	2.21	<0.0001
	10	1.13	0.3784	0.94	0.9175	1.57	0.9175	2.56	<0.0001	0.33	<0.0001	—	—	2.02	<0.0001
48	2	1.31	0.0025	0.52	<0.0001	0.64	<0.0001	1.53	<0.0001	0.14	<0.0001	1.88	<0.0001	0.80	0.0694
	4	0.85	0.2531	0.49	<0.0001	0.91	<0.0001	10.87	<0.0001	1.14	0.3116	9.34	<0.0001	1.37	0.0003
	6	2.25	<0.0001	0.64	0.0004	1.91	0.0004	4.98	<0.0001	2.45	<0.0001	9.31	<0.0001	1.35	0.0006
	8	2.29	<0.0001	0.76	0.024	1.64	0.024	5.13	<0.0001	1.08	0.7872	8.53	<0.0001	0.76	0.024
	10	2.12	<0.0001	1.23	0.0323	1.55	0.0323	3.45	<0.0001	0.92	0.7872	—	—	1.57	<0.0001
72	2	1.35	0.0006	1.20	0.0753	4.40	0.0753	0.66	0.0009	0.53	<0.0001	1.90	<0.0001	1.02	0.9997
	4	1.71	<0.0001	1.16	0.2031	2.00	0.2031	0.97	0.9955	0.94	0.9175	3.00	<0.0001	1.47	<0.0001
	6	2.61	<0.0001	2.44	<0.0001	2.80	<0.0001	1.12	0.4533	1.23	0.0323	53.00	<0.0001	2.33	<0.0001
	8	2.23	<0.0001	2.62	<0.0001	2.00	<0.0001	0.95	0.9589	1.20	0.0753	4.50	<0.0001	2.77	<0.0001
	10	1.00	>0.9999	2.84	<0.0001	9.60	<0.0001	0.97	0.9955	0.93	0.8594	—	—	3.04	<0.0001

#### 3.1.4 DNA double chain break analysis

In comparison with the control group, there was a significant increase in the formation of H2AX foci following DNA double‐strand breaks in the A253 and NM2C5 cell lines at doses of 2–10 Gy, as well as in the FADU cell lines at doses of 2–8 Gy. This effect was observed in the human (parental) head and neck CSC line at doses of 2–10 Gy and in the human (parental) melanoma CSC line at doses of 4–10 Gy. Interestingly, DNA double‐strand breaks were not detected in the WI‐38 and WSS‐1 cell lines (Table 3).

**TABLE 3:** Fold changes in the amount of DNA double‐strand breaks detected by H2AX compared to the control group.

	A253		FADU		MN2C5		HNSC		MSC		WI‐38		WSS‐1	
Gy	FC	*p*	FC	*p*	FC	*p*	FC	*p*	FC	*p*	FC	*p*	FC	*p*
2	5.52	<0.0001	1.46	0.0005	2.09	<0.0001	1.63	<0.0001	1.20	1.20	0.71	0.0170	1.84	<0.0001
4	11.56	<0.0001	1.04	0.9819	2.90	<0.0001	1.35	0.0042	2.00	2.00	1.01	0.9998	1.26	0.0288
6	29.76	<0.0001	1.87	<0.0001	3.88	<0.0001	2.71	<0.0001	3.06	3.06	1.07	0.8200	1.85	<0.0001
8	15.6	<0.0001	1.87	<0.0001	5.97	<0.0001	4.46	<0.0001	4.35	4.35	1.12	0.4547	0.76	0.0493
10	9.96	<0.0001	0.61	0.0019	5.43	<0.0001	4.40	<0.0001	6.72	6.72	—	—	0.48	0.0002

#### 3.1.5 Cell cycle analysis

It was observed that the A253 cell line exhibited cell cycle arrest at the G0/G1 or G2/M phases compared to the control group at doses of 2–10 Gy after 24 and 72 h. Similarly, the FADU cell line showed cell cycle arrest at the G2/M phase compared to the control group at doses of 2–10 Gy after 24 and 72 h. In the NM2C5 cell line, cell cycle arrest was observed at the G2/M phase at doses of 2–10 Gy after 24 and 48 h, and interestingly, at the S phase after 72 h. Both the human (parent) melanoma CSC line and the human (parent) head and neck CSC line exhibited cell cycle arrest at the G2/M stage at doses of 2–10 Gy after 24 and 72 h. No significant difference was found in the cell cycle of the WI‐38 cell line compared to the control group at doses of 2–10 Gy after 24 and 72 h. The WSS‐1 cell line showed differences in the G0/G1, S, and G2/M phases of the cell cycle compared to the control group at doses of 2–10 Gy after 24 and 72 h.

### 3.2 Comparison of cell survival response of non‐uniform and uniform areas

The effective dose of 4 Gy was determined for the cancer cell models, and the cancer cells were exposed to this dosage in partially and fully open 25 cm^2^ flasks. A comparison of cell survival responses between non‐uniform and uniform areas was conducted using the clonogenic assay method.

#### 3.2.1 Tumor cell models

In the A253 cell line, the coating effectiveness and survival rates were 0.11% and 37.04%, respectively, after exposure to fractionated 4 Gy. They were 0.12% and 38.89% after exposure to non‐fractionated 4 Gy. However, the difference was not significant (*t* = −0.378, *p* = 0.742). Similarly, in the FADU cell line, the coating effectiveness and survival rates were 2.89% and 57.78% following fractionated 4 Gy treatment, and 2.11% and 42.22% after non‐fractionated 4 Gy exposure. Again, the difference was not significant (*t* = 1.139, *p* = 0.373). In the NM2C5 cell line, the coating effectiveness and survival rates after fractionated 4 Gy exposure were 0.87% and 7.74%, respectively, while they were 3.4% and 30.36% after non‐fractionated 4 Gy exposure. The difference was significant (*t* = −0.79, *p* = 0.01). This indicated a 22.62% decrease in survival following fractional irradiation in the melanoma cell line NM2C5. In summary, it was observed that the fractional irradiation technique improved cytotoxicity in the melanoma cell model, but it did not produce a significant difference in the head and neck tumor models (Figure 3).

#### 3.2.2 Stem cell models

In the human (parental) head and neck CSC line, the coating efficiency and survival rates were 70.42% and 83.17% after fractionated 4 Gy, and 68.08% and 80.41% after non‐fractionated 4 Gy, respectively. However, the difference was not significant (*t* = 0.281, *p* = 0.805). In the human (parental) melanoma CSC line, no colonies were detected after fractionated 4 Gy treatment, resulting in a 0% survival rate. After non‐fractionated 4 Gy treatment, the coating efficiency and survival rates were 35.00% and 38.18%, respectively. The difference was highly significant (*t* = −29.12, *p* = 0.001). There was a 38.18% reduction in survival due to fractional irradiation in the melanoma stem cell line. Overall, it was observed that fractional irradiation increased cytotoxicity in the melanoma CSC model, while the survival effect of gradual irradiation was not observed in the CSC models, similar to the tumor cell models (Figure 3).

#### 3.2.3 Normal tissue cell models

In the WI‐38 cell line, the coating efficacy and survival rates were 0.50% and 50%, respectively, after exposure to fractionated 4 Gy, and 0.78% and 77.78% after exposure to non‐fractionated 4 Gy. The difference was significant (*t* = −45.62, *p* = 0.04). In the WSS‐1 cell line, the coating efficacy and survival rates were 2.67% and 11.11%, respectively, after fractionated 4 Gy exposure, and 3.67% and 15.28% after non‐fractionated 4 Gy exposure. However, the difference was not significant (*t* = −1.585, *p* = 0.254). Overall analysis of the data showed that fractional irradiation in the WI‐38 cell line resulted in a 27.35% reduction in survival. It was observed that the more radioresistant normal tissue cells (WI‐38) exhibited a higher level of cytotoxicity, similar to what was seen in the melanoma tumor and CSC model (Figure 3).

### 3.3 Irradiation model representing dose‐rate changes

The effective radiation dose of 4 Gy was established for the cancer cell models. Cancer cells were subjected to a dose rate of 50 MU/min (low‐dose rate), 300 MU/min (standard irradiation), or 600 MU/min (high‐dose rate) in 25 cm^2^ flasks. Cell survival responses at different dose rates were compared using the clonogenic assay method.

#### 3.3.1 Dose‐rate tumor cell models

**FIGURE 4:**
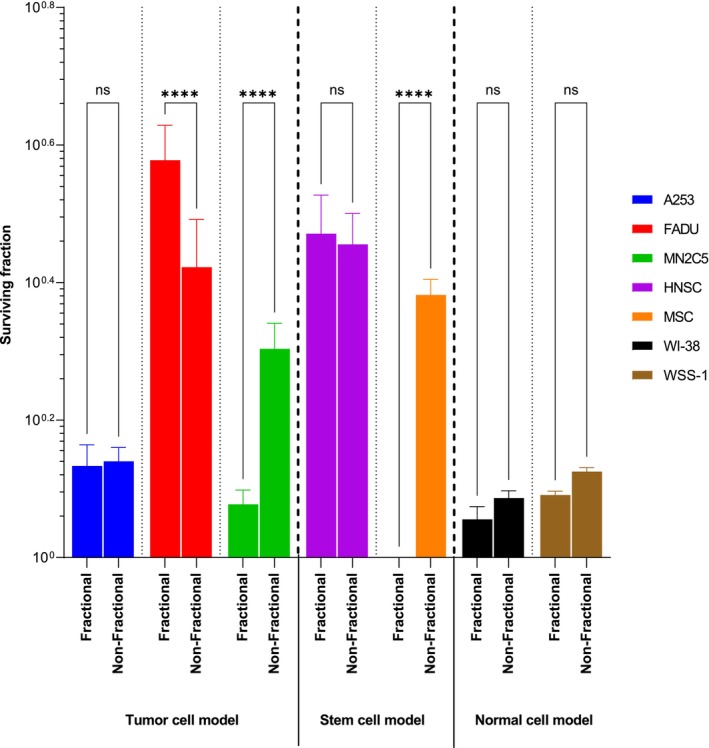
**T**he clonogenic assay results were evaluated across various dose rates following a 4 Gy radiation exposure in all cell models. The statistical analysis revealed that there were no significant differences denoted by “ns” (not significant) between the groups. However, an extremely significant difference (*p* < 0.0001) was observed, represented by “****”.

The survival rate following treatment in the A253 cell line at 4 Gy was 14.74% with a dose rate of 50 MU/min, 22.76% with a dose rate of 300 MU/min, and 12.5% with a dose rate of 600 MU/min. However, the difference was not significant (*t* = 0.281, *p* = 0.805). Similarly, in the FADU cell line, the survival rates at 4 Gy were 45.2% with a dose rate of 50 MU/min, 42.23% with a dose rate of 300 MU/min, and 34.32% with a dose rate of 600 MU/min. Again, the difference was not significant (*t* = 1.139, *p* = 0.373). In the melanoma cancer model, the survival rates after treatment at 4 Gy were 13.15% with a dose rate of 50 MU/min, 22.02% with a dose rate of 300 MU/min, and 51.02% with a dose rate of 600 MU/min. The difference was significant (*t* = −9, *p* = 0.02). It was observed that cytotoxicity decreased as the dose rate increased. When considering all the data from the tumor cell models, it was found that the cytotoxicity of head and neck tumor models increased with increasing dose rate, while the cytotoxicity of melanoma cell models decreased with increasing dose rate (Figure 4A).

#### 3.3.2 Dose rate stem cell models

The survival rate following treatment at 4 Gy in the human (parent) head and neck CSC line was 28.28% at a dose rate of 50 MU/min, 42.91% at a dose rate of 300 MU/min, and 33.24% at a dose rate of 600 MU/min. However, the difference was not significant. In the human (parental) melanoma CSC line, the survival rate after treatment at 4 Gy was 29.24% with a dose rate of 50 MU/min, 65.30% with a dose rate of 300 MU/min, and 43.33% with a dose rate of 600 MU/min. The difference was significant (*p* < 0.0001). Overall, the findings indicate that compared to standard dose‐rate treatments, cell survival decreased at high‐dose rates in head and neck stem cells, as expected. It was observed that low‐dose rates had a more negative effect on survival in melanoma stem cells compared to standard dose‐rate irradiation (Figure 4B).

#### 3.3.3 Normal tissue cell models

In the WI‐38 cell line, after treatment at 4 Gy, the survival rate was 8.76% with a dose rate of 50 MU/min, 22.22% with a dose rate of 300 MU/min, and 10.38% with a dose rate of 600 MU/min. However, the difference was not significant. Similarly, in the WSS‐1 cell line, the survival rate at 4 Gy was 12.28% after treatment with a dose rate of 50 MU/min, 10.09% with a dose rate of 300 MU/min, and 22.22% with a dose rate of 600 MU/min. Again, the difference was not significant. When the normal tissue cell models were analyzed collectively, it was observed that varied dose rates compared to standard irradiation had a more pronounced effect on cellular survival in the WI‐38 fibroblast cell line. Cell survival increased at low‐dose rates in the WSS‐1 epithelial cell line, but the expected survival effect was not observed at high‐dose rates (Figure 4C).

### 3.4 VMAT technique studies

#### 3.4.1 Tumor cell models

**FIGURE 5:**
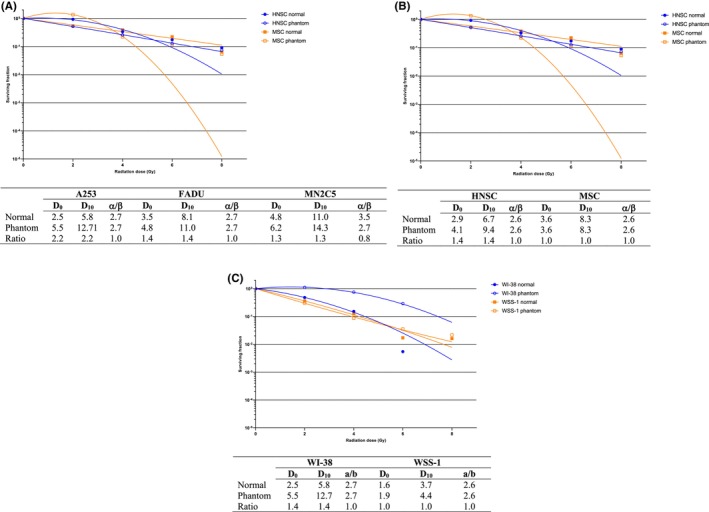
The cumulative results of clonogenic survival in tumor cell lines compared to normal tissue cell lines using phantom models. Three different models were utilized: (A) Tumor cell model, (B) stem cell model, and (C) normal tissue model. The clonogenic survival rates were measured and compared among these models to assess the relative effectiveness of the treatment on tumor cells versus normal tissue.

The A253 cell line exhibited a 20.38% decrease in survival following 2 Gy irradiation compared to the control group. A significant difference in viability was observed at low doses using the VMAT technique (*p* < 0.0001). In the FADU cell line, there was a 38.67% increase in survival after 2 Gy irradiation. However, this difference was no longer present at 4 Gy, and no significant difference in viability was detected using the VMAT technique (*p* = 0.06). In the melanoma cancer model, there was an 8.21% decrease in survival after 2 Gy irradiation compared to the control group. However, there was a 32.17% improvement in survival following high‐dose 6 Gy irradiation compared to the control group. In the NM2C5 cell line, significant differences in vitality were observed compared to the control group at 2 Gy, 6 Gy, and 8 Gy (*p* < 0.0001). Radiosensitivity in epithelial cell lines increased with the use of VMAT at 2 Gy, while radiosensitivity in the melanoma cell model increased at low doses and decreased considerably at doses above 4 Gy (Figure 5A).

#### 3.4.2 Stem cell models

The VMAT technique demonstrated a better effect on viability in the human (parental) head and neck CSC line, with a 40.76% decrease in survival observed after 2 Gy irradiation. This decrease in survival was found to be significant compared to the control group (*p* = 0.02). In the melanoma CSC line, there was an 81.87% decrease in survival at 2 Gy compared to normal cells. Radiosensitivity was observed to increase at higher dosages (*p* < 0.001), and it was also observed to increase at 2 Gy with the use of VMAT technique. Furthermore, the VMAT technique in the phantom was observed to enhance radiosensitivity and viability in the head and neck stem cell model, similar to the effects observed in tumor cell models. In melanoma stem cell lines, the VMAT technique exerted different effects at low and high doses (Figure 5B).

#### 3.4.3 Normal tissue cell models

In the WSS‐1 cell line, a radiation dose of 2 Gy led to a 5.6% decrease in survival. However, this reduction was not significant (*p* = 0.81). On the contrary, the WI‐38 cell line exhibited a 62.02% increase in survival compared to the control group after 2 Gy irradiation, and this difference was found to be significant (*p* < 0.0001). When the results of normal tissue cell models were analyzed collectively, it was observed that the VMAT technique reduced radiosensitivity in normal tissue cells, unlike in tumor and CSCs (Figure 5C).

## 4 DISCUSSION

Ionizing radiation can only have a biological effect in a living organism if the radiation energy is absorbed by living cells and tissues. The most significant target molecule in the emergence of the biological effects of radiation is DNA, as it contains genetic information and regulates growth, differentiation, and metabolism. DNA damage caused by ionizing radiation, either directly or indirectly, can lead to the repairable or irreparable damage of the cell, resulting in cell death or mutation. Therefore, one of the most important factors influencing radiation sensitivity and cell death is the cell's ability to repair DNA damage. This repair capability varies among healthy and cancer cell types.^12^ From a radiobiology perspective, cell death is typically defined as the loss of the cell's clonogenic capacity, which refers to the loss of its ability to generate new cells.^13^ When evaluating the radiobiological effectiveness of modern radiotherapy techniques, the lack of an experimental model that best reflects routine clinical practice is the most significant issue.^14^ It is recommended to perform radiobiological assessment of the physical dose delivered by IMRT as the dose distribution differs between IMRT and conventional radiation. The linear‐quadratic model overestimates the effects of high fractional doses of radiation. The biologically effective dose is particularly inaccurate for in vivo tumor responses since it does not account for reoxygenation. Improved models have been proposed for normal tissue responses, but the currently available models are not satisfactory for in vivo tumor responses, and better models should be proposed in future studies.^15^ The clinical applications of IMRT have been investigated, specifically evaluating the impact of dose rate associated with treatment duration. IMRT has the potential to reduce the doses delivered to normal tissues, thereby further minimizing the probability of complications arising from radiotherapy.^16^ VMAT techniques have several advantages in clinical practice. VMAT techniques provide enhanced coverage of the target volume, reduced treatment delivery time, and preservation of normal tissues when compared to conventional radiotherapy techniques.^17^ However, since all models are two‐dimensional, the extent to which these models represent daily clinical practice has been debated among researchers. The literature clearly highlights the need for advanced modeling and determination of safety margins for biological activity using in vivo experiences.

Ionizing radiation can only have a biological effect in a living organism if the radiation energy is absorbed by living cells and tissues. The most significant target molecule in the emergence of the biological effects of radiation is DNA since it contains genetic information and is a crucial molecule in growth, differentiation, and metabolism. Many enzymatic steps that follow DNA damage caused by the direct or indirect effects of ionizing radiation result in either a complete repair of the damage or leave the damage unrepaired, which results in cell death or mutation. Therefore, one of the most important factors affecting the radiation sensitivity and death of the cell is its ability to repair DNA damage. This ability varies between cell types. In terms of radiobiology, the concept of cell death is generally described as the loss of clonogenic capacity of the cell; the loss of a cell's ability to proliferate new cells [1, 2] The main issue, in light of all the research examining the radiobiological efficacy of modern radiotherapy techniques, is the lack of an experimental model that most accurately reflects routine clinical practice. Blockhuys et al. outlined the radiobiological questions with the IMRT approach and described these issues in a literature study in 2010 [3]. Sterzing et al. presented novel models for in vitro studies of IMRT and demonstrated the effect of IMRT on cellular survival in two different cell types [4]. The effect of prolonged radiotherapy treatment on cellular survival has been shown, but the techniques that shorten the time by altering dose rate in therapy were not fully understood. The “step and shot technique”, the first clinical application of IMRT, was investigated by Butterworth et al and the effect of dose rate related to extended treatment duration was evaluated in three different cell types. The modeling was applied as a segmental dose distribution to the monolayer flask. It was demonstrated using a simplified model of the daily applications that the length of time had an impact on cellular survival [5]. However, new volumetric techniques entered clinical use before the same group was able to adapt the results of 2010 paper into clinical practice. The same group started to use a model for VMAT in 2013. The in vitro results of both research indicate that different tumor cell types have different rates of cellular survival [6]. However, all of the models were two‐dimensional, and to what extent these models represented daily applications was debated by researchers as well. The literature makes clear the necessity for improved modeling and determination of the biological activity's safety boundaries using in vivo experiences.^18^

### 4.1 Results of standard irradiation technique

The response of cells to radiation is influenced by various factors. The distinct reactions of individual cells to radiation form the basis for tumor treatment using radiotherapy. The variation in radiation sensitivity among different tumor and normal tissue cells leads to specific dose limitations and clinical applications tailored to each tumor type. While it is acknowledged that in vitro studies have limitations in terms of representing real‐world applications as they do not consider the tumor microenvironment, clonogenic assays are widely accepted as the optimal model for assessing radiation sensitivity in vitro.^19^

In this study, we aimed to assess the cellular sensitivity in daily conventional irradiation techniques using various cell models, including tumor cells (head and neck and melanoma cancer cells), normal tissues (lung epithelium and fibroblast cells), and CSC models (head and neck and melanoma CSCs). Upon reviewing the collective results, it was observed that the radiosensitivity differed among the various tumor cells, normal tissue cells, and CSCs. Notably, the radiosensitivity of CSC models was found to be lower compared to tumor cell models. CSCs have been shown to exhibit increased resistance to radiation compared to non‐CSC cells, and their presence is believed to contribute to treatment failure and tumor recurrence.^3^, ^6^, ^20^ Several studies have consistently shown the resistance of CSCs to radiation. In a specific study focusing on breast CSCs, the cellular radiation response was examined in both monolayer cultures (MCF‐7) and mammospheres (MCF‐7S). It was observed that a CSC‐like population with the phenotype CD44+ CD24−/low exhibited relative radioresistance when comparing survival fractions.^8^ It has been discovered that the MCF‐7S cell line, which displays characteristics of CSCs, exhibits higher cell survival rates and lower levels of free radical production following radiation exposure. CSCs employ various mechanisms to acquire resistance to radiation. These mechanisms include DNA break repair, activation of cell cycle checkpoints, extension of the G2 phase, elimination of free radicals generated by irradiation, activation of self‐renewal pathways, self‐renewal capability, and evasion of cellular senescence.^6^, ^21^ The findings of our study were consistent with previous studies reported in the literature.

### 4.2 Comparison of cell survival responses of non‐uniform and uniform areas

The multi‐leaf collimator (MLC) system is employed in daily radiotherapy applications to deliver radiation to the target at different intensities and at different times, enabling the modification of dose intensity in both tumors and normal tissues and achieving desired dose distributions. This technique, known as IMRT, involves delivering the radiation beam as a cumulative sum of segmented irradiation to the tumor and surrounding normal tissues throughout the treatment course. Interplay between intercellular signaling systems and this segmented irradiation could influence the cellular response, potentially leading to either an increase or decrease in radiation sensitivity.^22^

The application of IMRT has provided valuable insights into its physical characteristics, such as its ability to deliver high doses to the target while minimizing radiation exposure to nearby critical organs. This capability has enabled the successful treatment of patients with complex‐shaped tumors, surpassing the limitations of traditional radiation therapy. Nevertheless, it should be noted that most IMRT techniques involve the delivery of treatment fractions over extended time periods and at lower dose rates. The radiobiological implications of reduced dose rates and fractionated irradiation techniques are not yet fully elucidated, and further research is needed to comprehensively understand their impact on treatment outcomes.^5^ Moreover, it is still uncertain whether IMRT accurately reflects the biological response. The majority of studies demonstrating the effectiveness of segmented irradiation are based on two‐dimensional MLC systems, similar to the one utilized in our study, despite the existence of various technological advancements documented in the literature. Therefore, further investigations are required to assess the biological implications of different IMRT techniques and their compatibility with the actual clinical scenarios.^5^, ^8^, ^21^, ^23^, ^24^

In our study, we investigated the effects of two‐dimensional fractionated irradiation techniques, representing both uniform and non‐uniform irradiation patterns, on the survival curve's 4 Gy shoulder region as the test dose. Following the completion of cell clonogenic survival studies, the findings revealed that the fractionated irradiation approach resulted in increased radio‐cytotoxicity and radiosensitivity in both melanoma and melanoma stem cell models. To the best of our knowledge, this is the first study to demonstrate that segmental irradiation enhances the radio‐cytotoxicity of melanoma cells. While long‐term therapy involving segmental irradiation has been reported to decrease radiosensitivity in various tumor types, it is noteworthy that our study observed an increase in radiosensitivity.

### 4.3 Irradiation model representing dose‐rate variations

The biological response to radiation is influenced by factors such as radiation energy, dose, and dose rate.^25^ Previous studies on dose rate effects have shown that lower dose rates result in less cytotoxicity, while higher dose rates lead to increased cytotoxicity. Lower dose rates may be associated with reduced cellular survival due to the prolonged treatment duration, as it allows more time for cellular repair mechanisms to counteract radiation damage. However, modern radiotherapy treatments have effectively addressed this issue by optimizing treatment lengths. When considering the data from tumor cell models collectively, cytotoxicity increased with higher dose rates in head and neck tumor models, whereas cytotoxicity increased with lower dose rates in the melanoma cell model. In the evaluation of normal tissue cell models as a whole, different dose rates were found to be more effective in terms of cellular survival compared to standard irradiation. Specifically, in the WI‐38 fibroblast cell line, cell survival was higher with different dose rates. In the WSS‐1 epithelial cell line, cell survival increased at low‐dose rates, but the expected survival effect was not observed at high‐dose rates. Consistent with our findings, Slosarek et al. also reported increased toxicity at low‐dose rates in both normal tissue cells and tumor cells. They suggested that low‐dose rates can have detrimental consequences, particularly in dynamic radiation procedures.^26^ This phenomenon, known as low‐dose hypersensitivity, is particularly distinct in melanoma cells. Overall, the response to radiation dose rates varies among different cell types and can have implications for treatment outcomes. Understanding the interplay between dose rate and cellular response is crucial for optimizing radiotherapy protocols and achieving optimal therapeutic efficacy while minimizing adverse effects.^23^ Another study conducted by Todorovic et al. further supports the phenomenon of “low‐dose hypersensitivity”. They observed this effect in isogenic radioresistant cells, but not in the parental cells. The authors attribute this phenomenon to its impact on DNA damage signaling pathways. Their findings provide additional evidence for the complex relationship between radiation dose, cellular response, and DNA damage signaling mechanisms, emphasizing the importance of understanding these processes for optimizing radiotherapy strategies.^27^

In this study, the efficacy of dose rate was evaluated in CSC models, marking the first instance in the literature. Interestingly, it was observed that lower dose rates had a more detrimental impact on survival compared to standard dose‐rate irradiations. It is advisable to consider these variances for therapeutic advantages in clinical practice and verify them through appropriate clinical protocols.

### 4.4 Results of VMAT irradiation technique

In this paper, we employed a cylindrical water phantom that is compatible with a reproducible irradiation system to investigate the impact of complex dose distributions obtained through IMRT techniques on cellular response. This system created physical conditions that are in line with the VMAT technique. However, determining the optimal experimental design that closely resembles daily clinical practice is not straightforward. Various researchers have proposed solid phantom models in the literature for this purpose.^4^, ^28^ This study is the first to utilize a water phantom. Upon evaluating the tumor tissue cell models collectively, it was observed that radiosensitivity increased with VMAT in all cell lines at 2 Gy, while the radiosensitivity decreased after 4 Gy in the melanoma cell model. It is hypothesized that radioresistant tumor cells exhibit an elevation in radiobiological activity as the dose per fraction is increased. In this study, which demonstrated an augmented radiosensitivity through low‐dose rate and segmental irradiation, the negative impact of VMAT was attributed to uncontrolled high‐dose rates at higher doses. When assessing outcomes that best represent daily clinical practice, it is imperative to reevaluate the application of stereotactic radiotherapy, which modifies the dose rate and segmental irradiation at high rates, both in terms of physical and radiobiological aspects, specifically for melanoma cells.

In volumetric irradiation techniques such as the VMAT technique, high doses are physically delivered to the tumor tissue, resulting in sharp dose gradients in normal tissues. While a high dose is maintained within the tumor, reduced dose regions are applied to normal tissues. One of the crucial radiobiological considerations associated with these new physical parameters is that irradiation can cause side effects in normal tissues beyond the expected levels. Dose constraints for normal tissues have been established based on clinical observations obtained from three‐dimensional radiotherapy applications.^8^, ^21^ The existing dosage restrictions may result in side effects within the therapeutic window in clinical settings, as different radiation techniques can enhance radiosensitivity and reduce cell survival. Our investigation revealed that the VMAT approach decreased radiosensitivity, leading to delayed side effects, particularly in the WI‐38 fibroblast cell line. This finding suggests the possibility of reducing delayed side effects or increasing dose limits. However, no significant difference was observed in the epithelial cell line, which represents the initial side effects of radiotherapy, when using VMAT irradiation techniques. In conclusion, the findings of this study should be validated through in vivo testing before being integrated into the current treatment regimens of clinical practice.

In conclusion, the findings of this study need to be validated through in vivo testing before incorporating them into the current treatment regimens of clinical practice. It is essential to consider the physical and radiobiological aspects of applying stereotactic radiotherapy, especially for melanoma cells, which involves modifying the dose rate and segmental irradiation at high rates. By doing so, we can better understand and manage the potential side effects in normal tissues and optimize treatment outcomes for patients.

## AUTHOR CONTRIBUTIONS

**Serra Kamer:** Funding acquisition (lead); investigation (equal); methodology (equal); resources (equal); supervision (equal); validation (equal); writing – review and editing (supporting). **Sunde Yilmaz Susluer:** Methodology (equal); validation (equal); writing – original draft (lead); writing – review and editing (lead). **Tugce Balci Okcanoglu:** Methodology (equal); validation (equal). **Cagla Kayabasi:** Methodology (equal); validation (equal). **Besra Ozmen Yelken:** Methodology (equal); validation (equal). **Sinan Hoca:** Investigation (equal); methodology (equal); visualization (equal). **Emin Tavlayan:** Investigation (equal); methodology (equal); visualization (equal). **Nezahat Olacak:** Conceptualization (equal); investigation (equal). **Yavuz Anacak:** Conceptualization (equal); supervision (equal). **Murat Olukman:** Conceptualization (equal); supervision (equal). **Cumhur Gunduz:** Investigation (equal); methodology (equal); supervision (equal); validation (equal).

## CONFLICT OF INTEREST STATEMENT

The authors have no conflicts of interest to declare.

## ETHICAL APPROVAL STATEMENT

No ethical approval statement required.

## Data Availability

Not applicable.
